# Micro-RNA miR-542-3p suppresses decidualization by targeting ILK pathways in human endometrial stromal cells

**DOI:** 10.1038/s41598-021-85295-2

**Published:** 2021-03-30

**Authors:** Xinlan Qu, Yuan Fang, Siying Zhuang, Yuanzhen Zhang

**Affiliations:** 1 Department of Obstetrics and Gynecology, Zhongnan Hospital of Wuhan University, Wuhan, 430071 China; 2 Department of Thyroid and Mammary Gland Surgery, Zhongnan Hospital of Wuhan University, Wuhan, China; 3 The Reproductive Medicine Center, Zhongnan Hospital of Wuhan University, Wuhan, 430071 China

**Keywords:** Cell biology, Molecular biology, Health care

## Abstract

Decidualization of human endometrial stromal cells (HESCs) is a vital step for successful pregnancy. However, the process by which micro-RNAs (miRNAs) regulate decidualization remains elusive. Our current study was designed to identify the mechanism of miRNA miR-542-3p and its potential targets in regulating decidualization. The results showed that miR-542-3p was down-regulated in HESCs. Luciferase assay confirmed that integrin-linked kinase (ILK) is a direct target of miR-542-3p. Overexpression of miR-542-3p resulted in decreased ILK and downstream transforming growth factor β1 (TGF-β1) and SMAD family member 2 (SMAD2) expression. Additional expression of ILK attenuates the miR542-3p-induced down-regulation of TGF-β1 and SMAD2, changes properties such as suppression of proliferation and invasion, and induction of apoptosis, thereby affecting the differentiation of HESCs. Moreover, miR-542-3p overexpression caused down-regulation of the angiogenic factors vascular endothelial growth factor (VEGF), cyclooxygenase-2 (COX-2) and matrix metalloproteinase-9 (MMP-9), and the supernatant of HESCs overexpressing miR-542-3p inhibited the formation of tubular structures in human umbilical vein endothelial cells (HUVECs), suggesting that miR-542-3p inhibits angiogenesis of HUVECs. Furthermore, in our mouse model, following injection of miR-542-3p mimic into the endometrium of mice at pregnancy day 8 (D8), we found decreased miR-542-3p expression and loss of embryo implantation sites. In conclusion, miR-542-3p can affect the process of endometrial decidualization by down-regulating ILK. The present study adds further understanding of the process and regulation of decidualization.

## Introduction

Embryo implantation refers to a continuous and complex dynamic biological process in which an activated blastocyst comes into contact with, and interacts with, the receiving endometrium. Implantation failure is the leading cause of infertility; however, the causes of implantation failure still remain unresolved. Although many potential causes of human infertility can be overcome through in vitro fertilization and embryo transfer techniques, the successful implantation rate remains low, at approximately 25%^1^. One of the main reasons for implantation failure is due to endometrial decidualization disorders. In order to successfully implant the blastocysts into the endometrium, endometrial stromal cells should have already begun to proliferate and differentiate, transforming into large, round, cytoplasm-rich, multinucleated decidual cells (i.e. decidualized endometrial stromal cells). Decidualization starts around the blood vessels, and then gradually expands to encompass the entire uterine cavity, causing extensive changes in the endometrium. In humans and mice, the extent of decidualization influences the degree of trophoblast invasion in later stages, playing an important role in the regulation of blastocyst implantation, placenta formation, and the maintenance of normal pregnancy^2^. Endometrial decidualization disorders may be the underlying reason for a variety of abnormalities in early pregnancy, such as recurrent abortion, infertility, and preeclampsia.

A number of genes and proteins are known to be involved in the regulation of decidualization, but the specific molecular mechanisms of initiation, establishment and maintenance of decidualization are not clear. Therefore, there is a need to research and explore the molecular mechanisms of decidualization from multiple perspectives and to identify key regulatory molecules involved in the process.

The most important molecules involved in gene regulation are micro-RNAs (miRNAs), which are endogenous non-coding RNA fragments found in eukaryotes functioning by binding to the 3′ untranslated region (3′UTR) of their target mRNA. Upon binding, miRNAs can inhibit expression of the target gene, thereby affecting various biological processes such as cell differentiation, development, apoptosis and proliferation. These small miRNA molecules are stable, specific and sensitive, and are the main biological regulators of many fundamental physiological processes including reproduction. Therefore, miRNAs are ideal biomarkers which may be utilized in the early diagnosis of many diseases^3,4^. Recent studies have found that miRNAs also play an important role in the regulation of pregnancy with many of the genes and proteins involved in endometrial receptivity and decidualization^5–9^. However, studies on the mechanisms of miRNA regulation in the decidualization of endometrial tissue remain elusive.

Researchers have demonstrated that miR-542-3p functions as a tumor suppressor gene, and is implicated in the regulation of many malignant diseases including breast, ovarian, bladder, and esophageal cancers^10–15^. Studies have shown that miR-542-3p may inhibit the proliferation and differentiation of tumor cells by down-regulating the protein expression of survivin^12^. In addition, miR-542-3p has been shown to inhibit tumor cell invasion by targeting AKT and bone morphogenic protein (BMP) signaling pathways^13,16^. Other studies^17,18^ have shown that miR-542-3p can directly act as a negative regulator of angiopoietin-2, mediating an anti-angiogenic effect. Qiao et al.^14^ recently demonstrated that miR-542-3p inhibits ILK/TGF-β1 signaling pathways in oral cancer. A study by Tochigi et al.^19^ suggested that miR-542-3p may be related to endometrial decidualization, while the specific mechanism is not clear. Overall, there is little research on the regulatory mechanisms of miR-542-3p related to endometrial decidualization, therefore, these processes are the focus of this study.

## Methods

### HESC culture, synchronization, and in vitro decidualization

Immortalized HESC lines were donated by the laboratory of Professor Haibin Wang of Xiamen University and cultivated according to the manufacturer’s instructions^20^. HESCs were cultured in Dulbecco's Modified Eagle's Medium and Ham's F-12 Nutrient Mixture (DMEM/F12, Sigma) with 10% charcoal stripped fetal bovine serum (CS-FBS, Biological Industries) in an atmosphere of 5% CO_2_ at 37 °C. Serum starvation was utilized overnight to synchronize HESCs in the G0/G1 phase. Decidualization in vitro was induced by incubating the HESC in DMEM/F12 with 2% CS-FBS containing 10 nM of estradiol (E2, Sigma), 1 μM medroxyprogesterone 17-acetate (MPA, Sigma), and 0.5 mM dibutyryl cAMP (db-cAMP, Sigma). The medium was changed every 48 h.

### Plasmid construction

The coding sequence (CDS) of ILK was cloned into a pcDNA3.1(+) vector (Invitrogen) to generate pcDNA3.1-ILK. The plasmid PUC57-ILK 3′UTR was extracted and digested with NotI and XhoI for 5 h, following which ILK 3′UTR was recovered. The pYr-MirTarget vector was extracted by enzyme digestion with 2 μl NotI for 5 h and 1 μl XhoI overnight. The fragment of ILK 3′UTR containing a putative binding site for miR-542-3p was inserted downstream of the luciferase gene in the pYr-MirTarget vector (Promega, Madison, WI, USA), designated the wild-type (WT) 3′UTR, using the following protocol: 1 μl T4 DNA Ligase, 2 μl 5 × T4 Buffer, 1 μl pYr-MirTarget, 6 μl ILK 3′UTR. The corresponding mutant construct was created by mutating the seed region of the miR-542-3p-binding site. The constructs were verified by sequencing. The transfections were performed with Lipofectamine 2000 reagent (Invitrogen; Thermo Fisher Scientific, Inc.) according to the manufacturer's protocol.

### Oligonucleotide transfection

Immediately prior to transfection, the culture medium containing HESCs was changed to antibiotic-free decidualization medium containing 0.5 mM 8-br-cAMP and 10^−6^ M MPA. HESCs were seeded in a 6-well plate and transfected with miR-542-3p mimics, miR-542-3p inhibitors, negative control (NC) and pCDNA3.1-ILK using Lipofectamine 2000 (Invitrogen, USA) according to the manufacturer’s instructions. 100 μl serum-free opti-MEM was used to dilute 5 μl siRNA. Then, 4 μl Lipofectaminetm 2000 was diluted in 100 μl opti-MEM, the mixture of Lipofectaminetm 2000 and siRNA diluent was mixed at room temperature for 5 min, and cells were cultured at 37 °C, 5% CO_2_ for 48 h. Following this process, HESCs were ready for the study.

### ELISA (enzyme-linked immunosorbent assay)

100 μl biotinylated antibodies of PRL and IGFBP-1 were used as markers for decidualization. ELISA (elabscience) was performed to measure PRL and IGFBP-1 in the HESC culture media according to the manufacturer’s protocol. The assay was performed in triplicate and normalized to reflect the total protein content of the cultures.

### RNA extraction and quantitative polymerase chain reaction (q-PCR)

Total RNA was extracted from cultured HESCs using TRIzol reagent (Aidlab), and 4.43 μg of total RNA was used to synthesize cDNA according to the manufacturer’s instructions. The q-PCR was performed using SYBR premix Ex Taq TM (Takara). The primer sequences are shown in Table 1. 2^−△△CT^ method was used to calculate relative mRNA expression. All experiments were repeated at least three times.

**Table 1:** Primers for q-PCR in our study.

Genes	Primers (5′–3′)
miR-542-3P	5′-GTCGTATCCAGTGCAGGGTCCGAGGTATTCGCACTGGATACGACTTTCAGTT-3′ 5′‑TGTGACAGATTGATAACTGAAA‑3'
U6	5′-CGCTTCGGCAGCACATATAC-3′ 5′-AAATATGGAACGCTTCACGA-3′
ILK	5′-TGTGGAGTTTTGCAGTGCTT-3′ 5′-CGCTTTGCAGGGTCTTCATT-3′
TGF-β1	5′-CAGCAACAATTCCTGGCGATACCT-3′ 5′-CGCTAAGGCGAAAGCCCTCAAT-3′
Smad2	5′-GTCTCCAGGTATCCCATCG-3′ 5′-TTAGGATCTCGGTGTGTCGG-3′
VEGF	5′-ATCCAATCGAGACCCTGGTG-3′ 5′-ATCTCTCCTATGTGCTGGCC-3′
MMP9	5′-CAGTCCACCCTTGTGCTCTTCCCTG-3′ 5′-ATCTCTGCCACCCGAGTGTAACCA-3′
COX2	5′-TACAATGCTGACTATGGCTACAAAA-3′ 5′-TGAAAAACTGATGCGTGAAGTGCTG-3′
GAPDH	5′-TCAAGAAGGTGGTGAAGCAGG-3′ 5′-TCAAAGGTGGAGGAGTGGGT-3′

### Western blot

Proteins were extracted from cultured HESCs using RIPA lysis buffer, and the concentration of protein was detected by the BCA Protein Assay Kit (Beyotime). Samples were then separated by 10% SDS-PAGE gels (Beyotime). The primary antibodies were applied according to the provided recommendations: anti-ILK (1:5000, Abcam), anti-TGFβ1 (1:200, Boster), anti-SMAD2 (1:1000, Affinity), anti-VEGF (1:200, Boster), anti-COX-2 (1:700, Proteintech Group), anti-MMP-9 (1:800, Abcam) and anti-GAPDH (1:1000, Abcam). Finally, positive bands were detected using the chemiluminescent ECL Plus reagent (Beyotime) according to the manufacturer’s protocol. The densitometry of bands was quantified using Quantity One version 4.6.0 software (Bio-Rad). Expressions of proteins were normalized to GAPDH protein.

### Cell proliferation assays

The proliferation of HESCs was evaluated by MTT assay (Sigma) according to the manufacturer’s instructions. HESCs were seeded on 96-well plates (5 × 10^3^/well) with miR-542-3p mimics, negative control oligonucleotides or pcDNA3.1-ILK vector, and cell numbers were counted at 24, 48 and 72 h after transfection. The experiments were repeated three times.

### Cell invasion assay

Cell invasion was examined using Transwell assay (BD Biosciences). Matrigel (Corning Glass Works) was dissolved overnight at 4 °C and then diluted with serum-free DMEM/F12 medium (1:3). Following serum starvation for 24 h, 200 μl cell suspension (4 × 10^5^) was seeded into the upper chamber. The lower chamber was filled with medium containing 10% FBS. After 48 h of culture, cells which had invaded through the membrane to the lower surface were fixed, stained, and photographed under microscopy. The mean number of cells was determined by selecting 5 fields per well for each group.

### Cell apoptosis assay

Flow cytometric analysis with Annexin V-APC/7-AAD Apoptosis Detection Kit (Nanjing Keygen Biotech. Co.) was performed to evaluate cell apoptosis. After transfection for 48 h, HESCs were harvested, trypsinized and incubated in 5 μl Annexin V-APC for 5–15 min. 5 μl 7-AAD was then added and the cells were analyzed with flow cytometry.

### Luciferase reporter assay

The putative miR-542-3p binding site in the ILK gene was identified at bases 233–240 of the 3′UTR by Target Scan (http://www.targetscan.org/vert_71). A 72 bp ILK mRNA fragment was amplified and cloned into the pYr-MirTarge construct (Yrgene) via NotI/XhoI. All sequences are listed in Table 1. Luciferase activity was measured with the dual luciferase reporter assay system (Byotime). HEK293 cells were cultured in DMEM supplemented with 10% FBS in 12-well plates and co-transfected with WT or mutant reporter vectors and miR-542-3p mimic, miR-542-3p inhibitor, mimic control (mimic NC) or inhibitor control (inhibitor NC). All of the constructed plasmids were verified by DNA sequencing. After transfection, the cells were harvested at 48 h and luciferase activity was detected and normalized to Renilla activity.

### Tube formation assay

The HESCs transfected with different treatments were seeded in 6-well plates at a concentration of 2 × 10^5^ cells/well. Following 24 h incubation, the supernatants were collected and centrifuged at 500×*g* at 4 °C for 5 min. HUVECs (American Type Culture Collection, Manassas) were seeded on a thin layer of Matrigel (BD Biosciences) that had been melted at 4 °C overnight and incubated at 37 °C for 30–60 min in pre-cooled 24-well plates. After 40 min incubation, HUVEC cells were resuspended with the HESC supernatant and incubated for 6 h at 37 °C. Three microscope fields were selected randomly and photographed. Tube forming ability was quantified by counting the total number of tube meshes and master segment length under a 10 × objective in four different fields per well. Each experiment was performed at least three times.

### Animals and treatment

Animal treatment and preparation were in accordance with the methods previously described^21^. Virgin CD-1 3-week-old female mice and male mice (HFK Biological Technology Co.) were housed in the Experimental Animal Center of Zhongnan Hospital, Wuhan University in a controlled environment (14 h light/10 h darkness), with adaptive feeding for one week. Ovulation was induced at 17:00 in female mice by intraperitoneal injection of 10 IU human menopausal gonadotrophin (HMG), followed by intraperitoneal injection of 7.5 IU human chorionic gonadotrophin (HCG) 48 h later. Females were then mated with untreated males in the proportion 2:1. The next morning, vaginal sperm plugs were checked, and the day on which a plug was observed was designated gestation day zero (D0). 30 of the pregnant mice were randomly divided into two groups and sacrificed on D0 and D8 (n = 15) and the endometrium was removed for inspection. Another 30 pregnant mice were randomly divided into three groups (n = 10). After anesthesia on Day 2 of pregnancy, the ovaries and uterus were exposed through a midline incision on the back. 20 μl miR-542-3p mimic, miR-542-3p mimic NC were injected into the uterine from the uterine horn near the ovary with a micro syringe, and the same amount of sterile PBS was injected into the uterine horn on the other side. In the sham operation group, cornual puncture was performed, but no drug was injected. Uteri were collected on Day 8 of pregnancy and the number of implanted embryos was counted. The protocol of the current study was approved by the Institutional Ethics Committee of Zhongnan Hospital of Wuhan University (2016056), and was carried out in accordance with approved guidelines.

### Statistical analysis

All data are presented using GraphPad Prism. The experimental data are represented as the mean ± standard deviation (SD) of at least three independent experiments. Statistical significance of differences between groups was determined by Student’s t test. Data from MTT analysis were analyzed by one-way analysis of variance (ANOVA) followed by Student–Newman–Keuls post-hoc test. Differences were considered significant if p < 0.05.

## Results

### Expression of miR-542-3p is decreased during decidualization of HESCs

**Figure 1:**
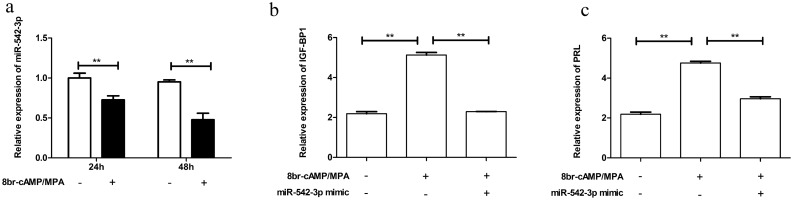
Relative expression of miR-542-3P and decidual marker (PRL, IGFBP-1) in HESCs during artificially induced decidualization. (**a**) The expression level of miR-542-3p was normalized to U6 in HESCs after treated with 8-br-cAMP and MPA for 24 h and 48 h. (**b**, **c**) The expression level of PRL, IGFBP-1(normalized to GAPDH) in HESCs after treated with 8-br-cAMP/MPA or miR-542-3p mimic were performed by ELISA. Significance is marked as **p < 0.01. Values are expressed as mean ± SD of three independent experiments.

To verify if mir-542-3p expression is altered with decidualization, HESCs were treated with cAMP and MPA. PRL and IGFBP-1 were used as markers of decidualization. Interestingly, miR-542-3p expression was found to decrease with decidualization in a time-dependent manner (Fig. 1a). When HESCs were transfected with miR-542-3p mimic, the expression of PRL and IGF-BP1 was decreased (Fig. 1b,c). We suspected that this down-regulation of miR542-3p may be closely related to decidualization.

### MiR-542-3p inhibits cell proliferation and invasion, and induces cell apoptosis

**Figure 2:**
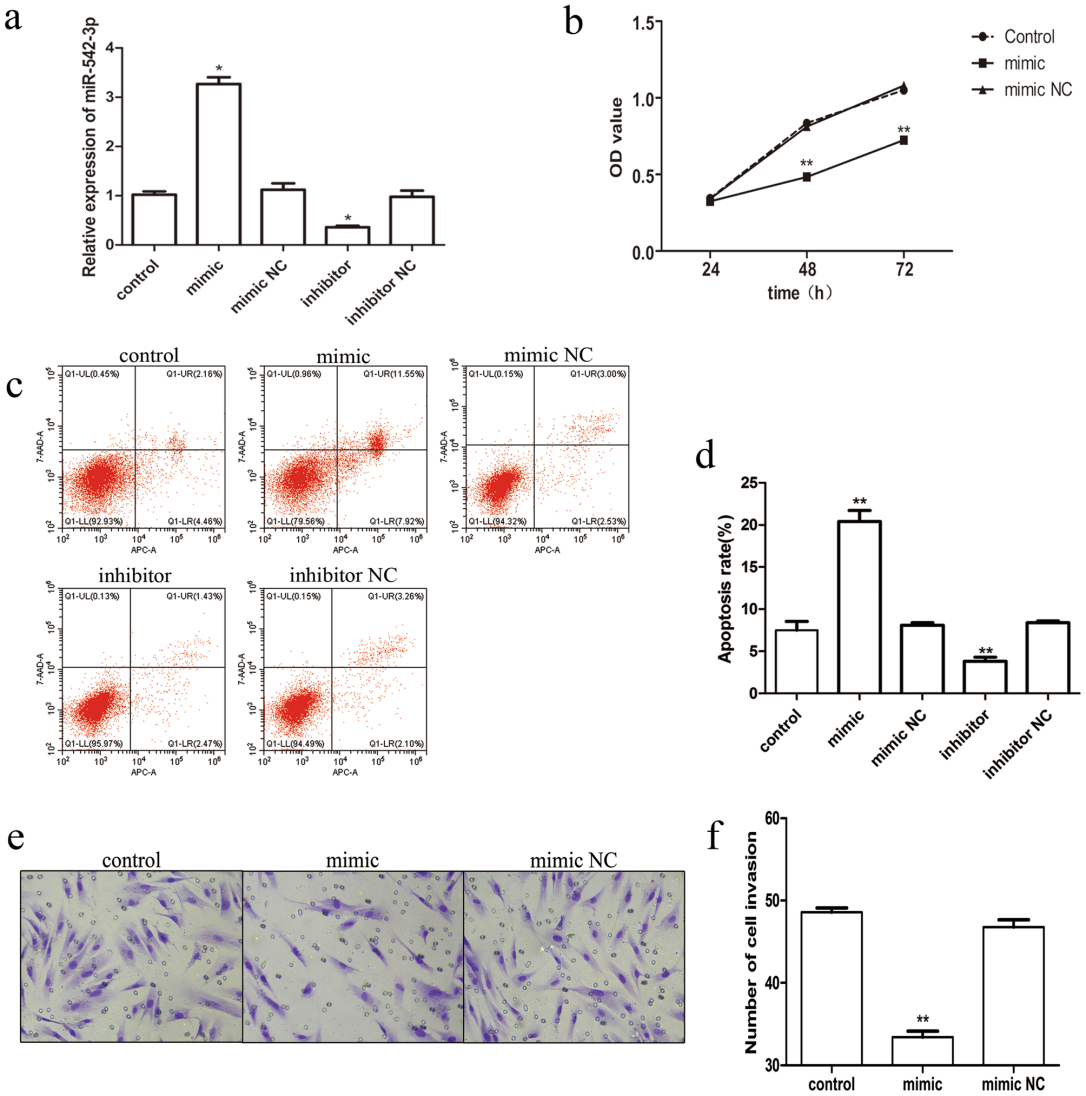
Effect of miR-542-3p overexpression on decidualizing HESCs. (**a**) q-PCR analysis of miR-542-3p levels in HESCs transfected with a miR-542-3p mimic or mimic NC or inhibitor or inhibitor NC and decidualized for 3 days. (**b**) MTT assay analysis of HESCs proliferation in control or miR-542-3p mimic or mimics NC groups and decidualized for 3 days. (**c**, **d**) Analysis of percent apoptosis of HESC cells by Flow cytometric analysis in control or miR-542-3p mimic or mimics NC or inhibitor or inhibitor NC groups and decidualized for 3 days. (**e**, **f**) Representative images (200X) of Transwell assay showing invasion ability of HESC from control or miR-542-3p mimic or mimics NC groups and decidualized for 3 days. Significance is marked as **p < 0.01. Values are expressed as mean ± SD of three independent experiments ILK as a miR-542-3p direct target.

Due to the down-regulation of miR-542-3p in HESCs, we speculated that miR-542-3p might have a negative role in the development of decidualization. To confirm the effects of miR-542-3p on HESC characteristics in vitro, cultured HESCs were transfected with miR-542-3p mimics or inhibitors. The RT-PCR results confirmed the validity of miR-542-3p mimics and inhibitors (Fig. 2a). MTT assay showed that overexpression of miR-542-3p significantly decreased the proliferation of HESCs (Fig. 2b). Flow cytometry was used to examine the rate of apoptosis of the HESCs transfected with miR-542-3P mimics or inhibitors. The number of apoptotic cells was found to be significantly higher in the miR-542-3P mimic group (Fig. 2c,d). Transwell assays were performed to evaluate cell invasion. The results showed that cell invasion capacity was significantly decreased when HESCs were transfected with miR-542-3p mimic (Fig. 2e,f). Taken together, these results indicate that miR-542-3p demonstrates a suppressive role in cell proliferation and invasion and an inductive role in cell apoptosis in vitro.

**Figure 3:**
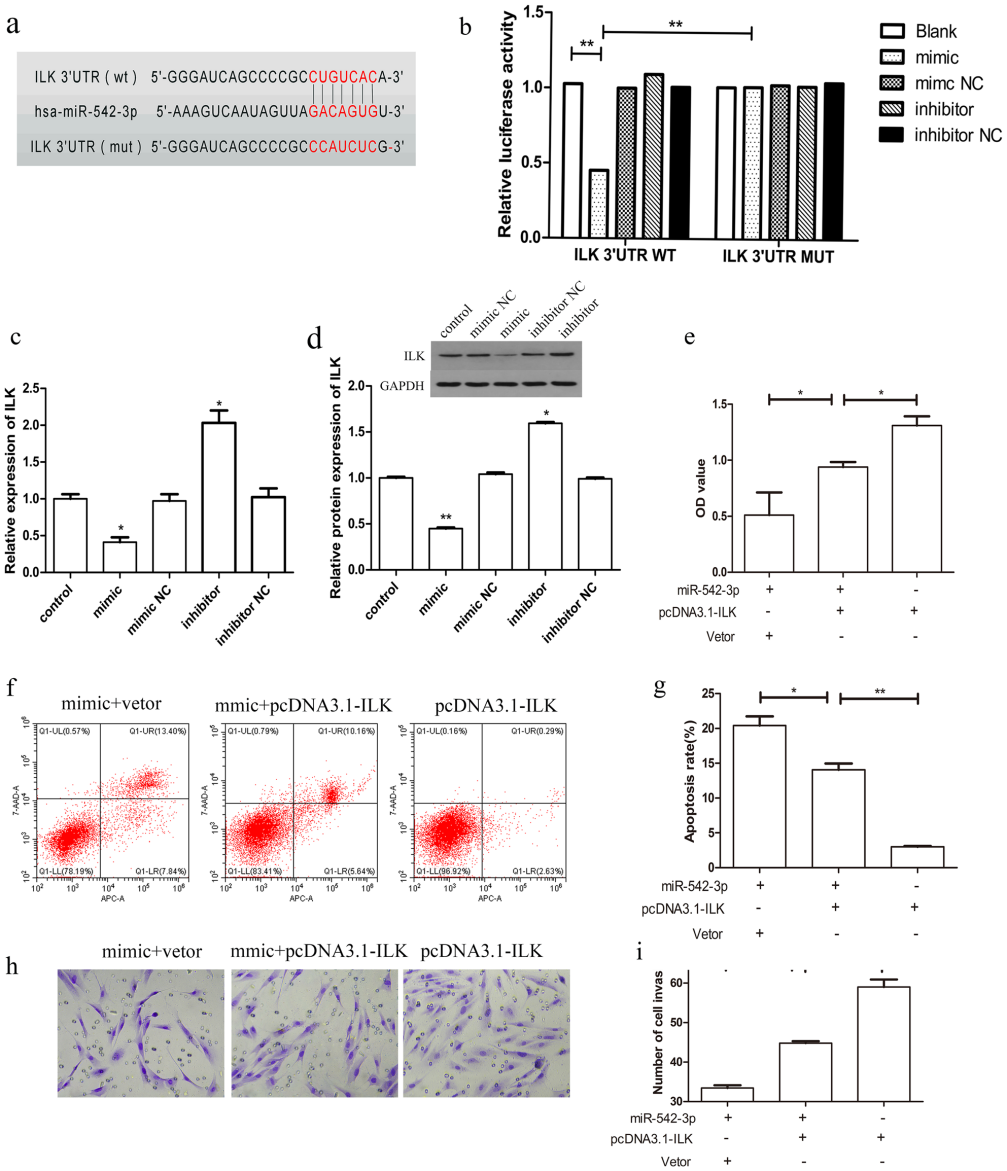
The confirmation of the miR-542-3p target ILK and effect of ILK-overexpression on cell Proliferation, invasion and apoptosis. (**a**) The predicted target sequence of miR-542-3p in the 3′-UTR of ILK and the mutated sequence are shown. (**b**) Relative luciferase activity of ILK-wt and ILK-mut reporter vectors co-transfected with miR-542-3p mimic or mimics NC or inhibitor or inhibitor NC miRNA in decidualized HESCs. Luciferase activity was measured and normalized by Renilla luciferase activity after 48 h. (**c**, **d**) q-PCR and western blotting analyzed the ILK expression in control or miR-542-3p mimic or mimics NC or inhibitor or inhibitor NC groups. (**e**) MTT assay analysis of HESCs proliferation after co-transfected with pcDNA3.1-ILK vector, together with/without miR-542-3p mimic. (**f**, **g**) Analysis of percent apoptosis of HESC cells by Flow cytometric analysis after co-transfected with pcDNA3.1-ILK vector, together with/without miR-542-3p mimic. (**h**, **i**) Representative images (200X) of Transwell assay showing invasion ability of HESC after co- transfected with pcDNA3.1-ILK vector, together with/without miR-542-3p mimic. Significance is marked as *p < 0.05, **p < 0.01.Values are expressed as mean ± SD of three independent experiments.

We aimed to reveal the mechanisms underlying the effects of miR-542-3p on HESC proliferation, invasion and apoptosis. Bioinformatic analysis was performed to identify the possible targets of miR-542-3p. As predicted by TargetScan and Pictar, ILK is a potential target of miR-542-3p. The 3′UTR of ILK containing the putative miR-542-3p binding site, which was predicted to be located at positions 233–240, was cloned for a luciferase reporter assay (Fig. 3a). The results showed that miR-542-3p overexpression inhibited the luciferase activity of WT, but did not inhibit the mutant 3′UTR ILK (Fig. 3b). Furthermore, overexpression of miR-542-3p suppressed mRNA and protein expression of ILK, with the opposite results of up-regulated expression of ILK during inhibition of miR-542-3p (Fig. 3c,d). These data suggest that ILK is a direct target of miR-542-3p.

### ILK is involved in miR-542-3p-induced suppression of cell proliferation and invasion, and promotion of cell apoptosis

We investigated if miR542-3p exerts its functions via regulating ILK. We used an ILK overexpression vector (pCDNA3.1-ILK) to enhance ILK expression in the miR-542-3p-overexpressing HESCs. As shown in our study (Fig. 3e–i), elevated ILK expression resulted in significantly greater cell proliferation and invasion, and stronger inhibition of cell apoptosis. These results imply that ILK reverses the suppressive effects of miR-542-3p in HESCs.

### Overexpression of miR-542-3p decreases the expression of ILK/TGF-β1/SMAD2 signaling pathways

**Figure 4:**
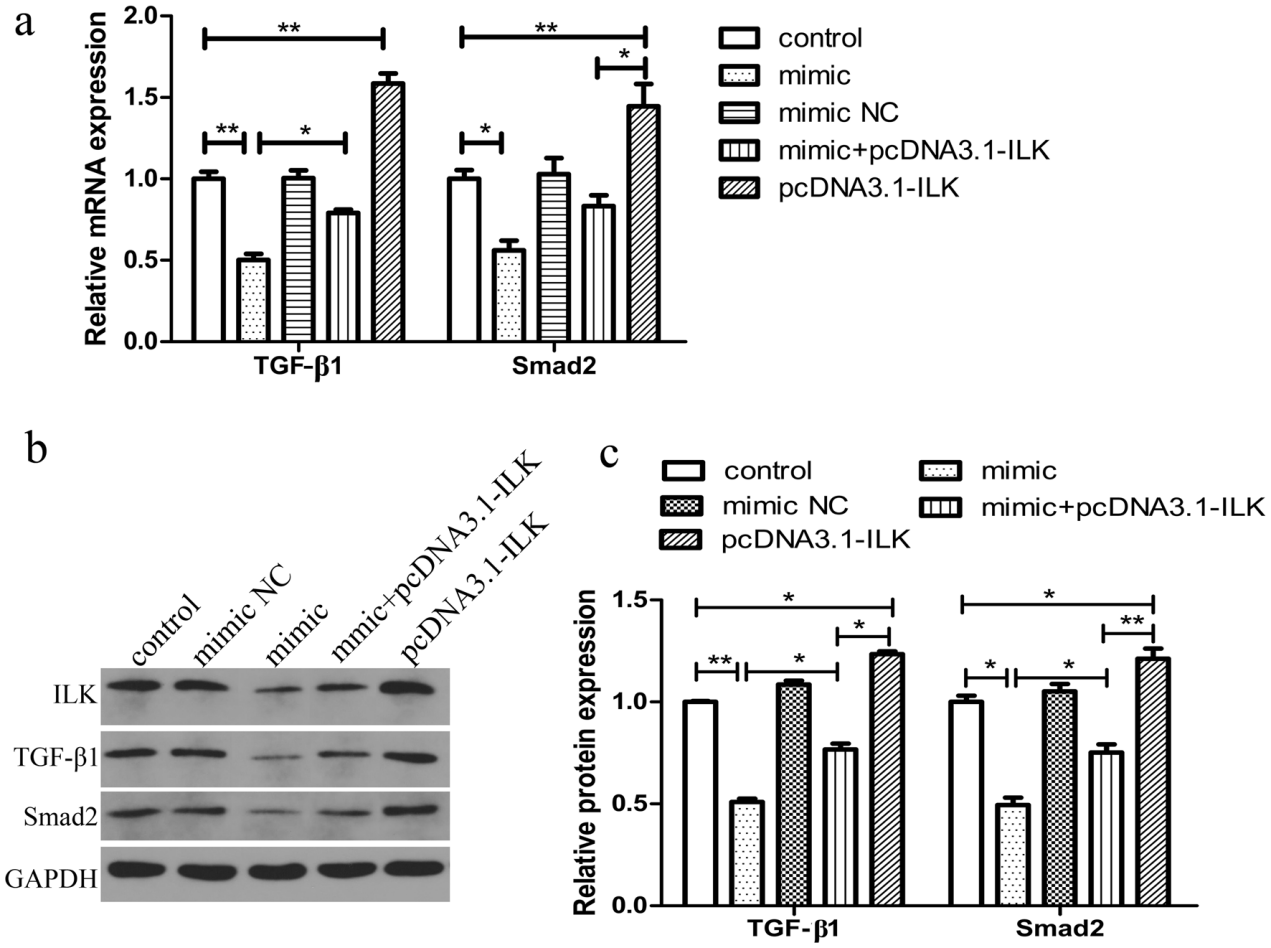
Effects of miR-542-3p and ILK on expression of TGF-β1/Smad2 pathway. (**a**) qRT-PCR analysis of TGF-β1and Smad2 mRNA levels. (**b, c**) Representative western blots showing TGF-β1 and Smad2 protein levels in HESCs cell after co-transfected with pcDNA3.1-ILK vector, together with/without miR-542-3p mimic or mimic NC. *p < 0.05, **p < 0.01. Values are expressed as mean ± SD of three independent experiments.

ILK may be involved in the regulation of cell proliferation, invasion and apoptosis by activation of the TGF-β1/SMAD2 pathway^22,23^, however, the exact mechanism of this process is unclear. Therefore, we examined the role of ILK in the inhibition of the TGF-β1/SMAD2 pathway. Compared with the control group, ILK mRNA and protein levels increased in the pcDNA3.1-ILK group and decreased in the miR-542-3p mimic group (Fig. 4a,b). Up-regulation of TGF-β1 and SMAD2 expression levels were found following overexpression of ILK in the pcDNA3.1-ILK group. Moreover, miR-542-3p also directly down-regulated TGF-β1 and SMAD2 expression levels (Fig. 4). Thus, these data indicate that miR-542-3p inhibits ILK protein expression, which is related to the expression of TGF-β1 and SMAD2. Hence, miR-542-3p inhibits the ILK/TGF-β1/ SMAD2 signaling pathway.

### MiR-542-3p decreases HESC angiogenesis in vitro

**Figure 5:**
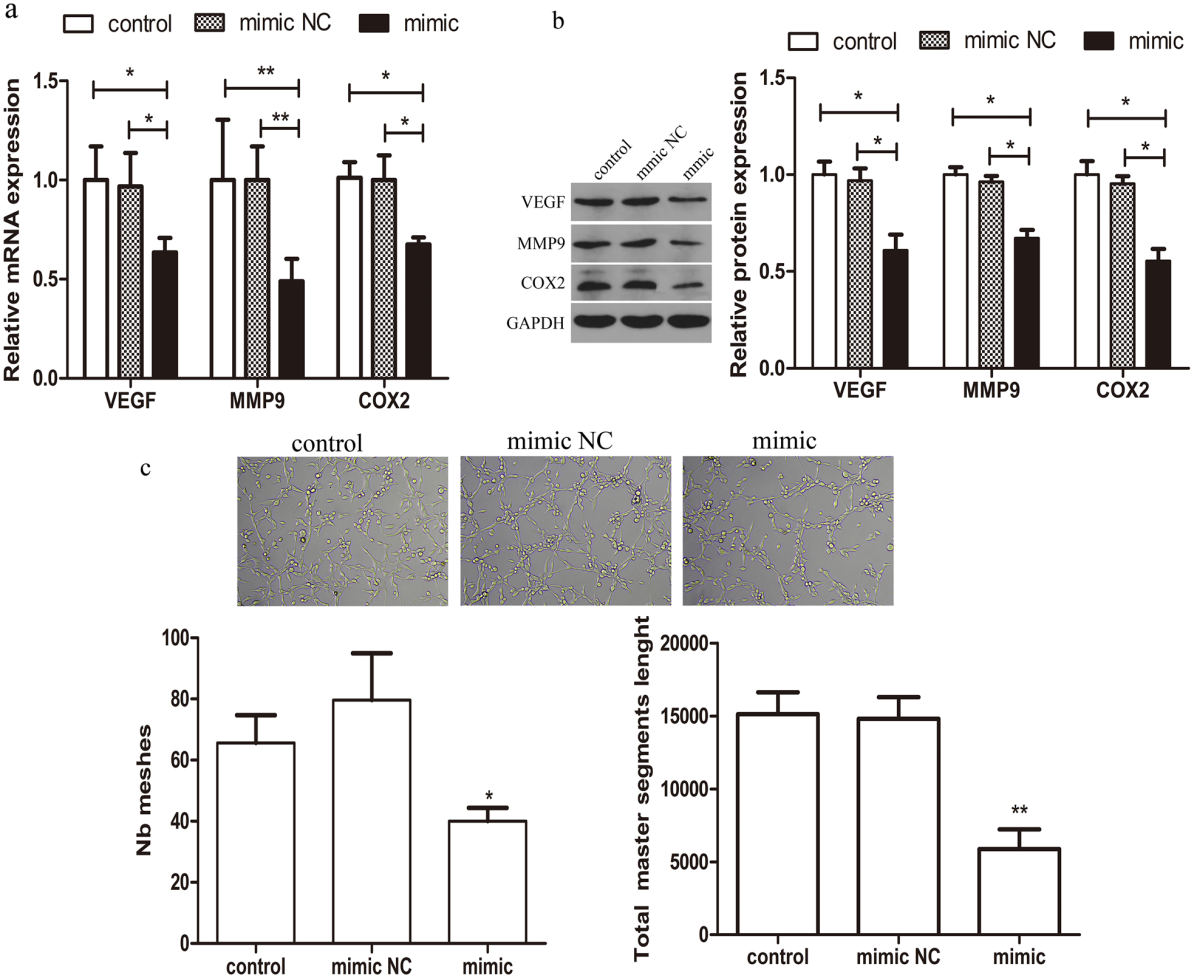
Effect of miR-542-3p overexpression on angiogenetic capacity of HESCs. (**a**) q-PCR analysis of VEGF, MMP9.COX2 mRNA levels in control or miR-542-3p mimic or mimics NC groups. (**b**) Representative western blots showing VEGF, MMP9.COX2 protein levels in control or miR-542-3p mimic or mimics NC groups. (**c**) Capillary-like tube formation performed by HUVECs with /without the supernatant of miR-542-3p-overexpressing HESCs. Representative images (100X) are showed. Number of Tube area (tube meshes and total master segment length) were acquired automatically. *p < 0.05, **p < 0.01. Values are expressed as mean ± SD of three independent experiments.

Angiogenesis is vital for endometrial receptivity and successful pregnancy. Elevated miR-542-3p and ILK expression have been found to be related to angiogenesis during tumorigenesis^24–26^. Here we explored the relationship between miR-542-3p and angiogenesis in HESCs during in vitro decidualization. The results showed that the expression of angiogenic elements VEGF, MMP-9 and COX-2 was gradually induced in HESCs during decidualization. Inhibiting miR-542-3p expression had the effect of reducing the expression of VEGF, MMP-9, and COX-2 (Fig. 5a,b). Moreover, an in vitro angiogenesis model of HUVECs was established for tube formation assay. Compared with control group, there was a reduction of tube meshes and total master segment length in HUVECs treated with supernatants from miR-542-3p overexpressing HESCs (Fig. 5c). These data indicate that miR-542-3p inhibits angiogenesis of HUVECs.

### MiR-542-3p leads to loss of embryo implantation

**Figure 6:**
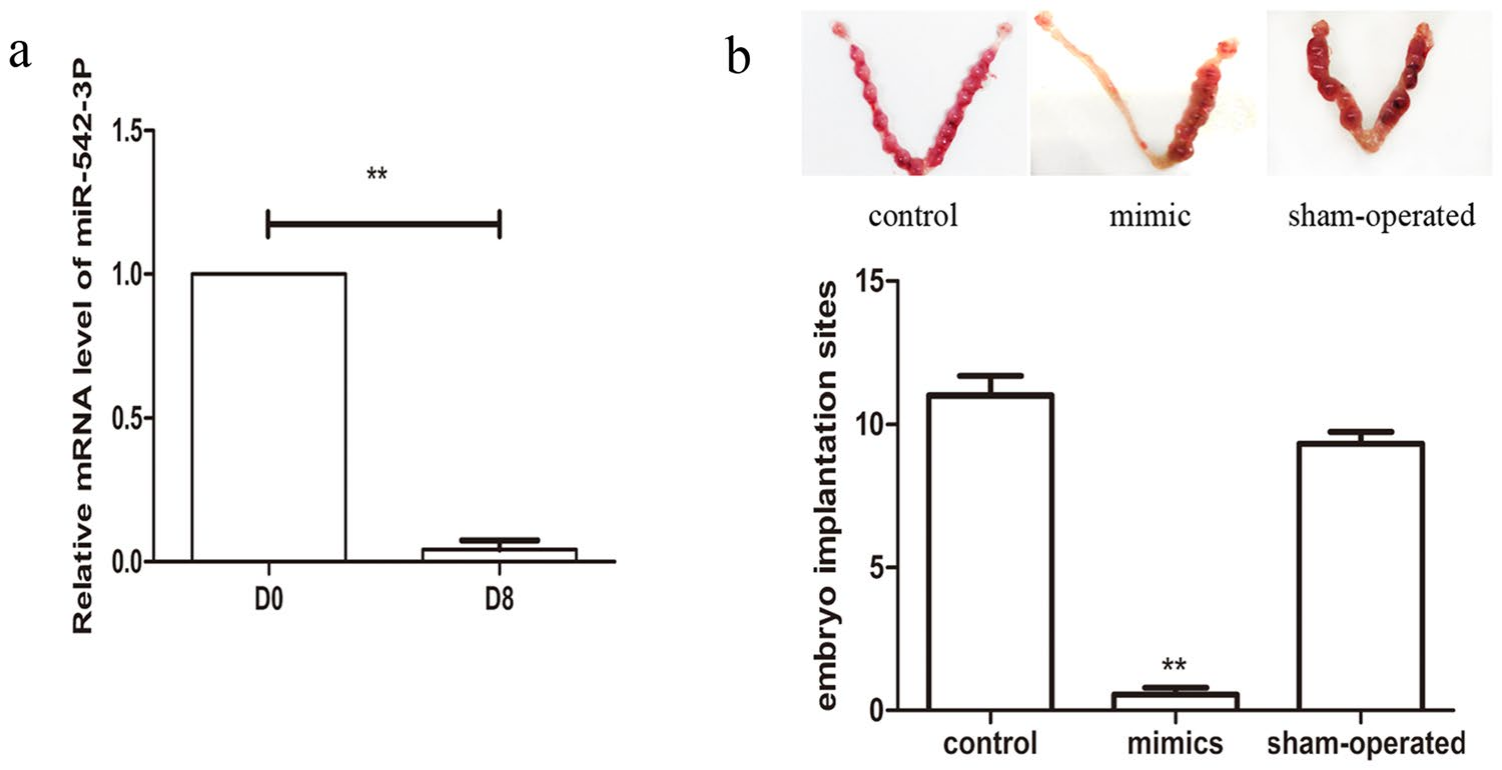
Relative mRNA level of miR-542-3p in early pregnancy and the effect of miR-542-3p overexpression on decidualization in mice uterus. (**a**) The mRNA level of miR-542-3p was analyzed by q-PCR on gestation D1 and D8. (**b**) Images showing embryo implantation sites on gestation D8 after injecting with miR-542-3p mimics unilaterally at the uterine horns or sham groups. Significant fold changes are marked by **p < 0.05 vs. D1. Values are expressed as mean ± SD, n = 15.

Furthermore, we established a mouse model and measured the expression levels of miR-542-3p on day 1(D1) and day 8 (D8) of gestation. D1 was designated as the day that vaginal sperm plugs were observed. Results showed that there was a down-regulation of miR-542-3p mRNA level in the mice endometrium on gestation D8 (Fig. 6a). Mice were then injected with miR-542-3p mimics unilaterally at the uterine horn to observe embryo implantation. It was found that there was a decreased rate of embryo implantation in mice treated with miR-542-3p mimics when compared to sham and control groups (Fig. 6b).

## Discussion

In our study, we provide evidence to support an important role for miR-542-3p in endometrial decidualization. MiR-542-3p was found to have a significant effect on HESC differentiation and angiogenesis. We focused on the regulation of miR-542-3p and its target, ILK signal pathway, which confirmed that miR-542-3p plays an important role in decidualization in vivo and in vitro through targeting ILK expression.

Previous studies on miR-542-3p expression have been mostly focused on tumorigenesis of various cells such as primary breast carcinoma, neuroblastoma, osteosarcoma, and colorectal cancer^27–30^. MiR-542-3p plays a pivotal role in tumor cell proliferation, migration, invasion and apoptosis, and has been shown to be useful as a prognostic marker for some cancers^30^. Embryo implantation is believed to be similar to tumorigenesis, however, there have been few studies conducted on the effect of miR-542-3p on embryo implantation, let alone research on decidualization. Estella et al.^31^ found 26 miRNAs which were up-regulated and 17 miRNAs which were down-regulated during decidualization. Among these miRNAs, miR-542-3p showed no significant change in endometrial samples. Tochigi et al.^19^ demonstrated that miR-542-3p directly targets IGFBP1 in HESCs and indirectly regulates the expression of PRL and WNT4. Another study^32^ confirmed that the overexpression of miR-542-3p could influence the capacity for migration and invasion of endometriotic cells in an ectopic environment through regulation of IGFBP1 expression. As previously reported, a single miRNA can influence hundreds of mRNAs and regulate a wide range of biological functions, including cell proliferation, migration, differentiation and apoptosis. In our study, down-regulation of miR-542-3p was observed in the decidualization group compared to the control group. An increase in miR-542-3p expression led to a decrease in expression of PRL and IGFBP-1, and promoted a noticeable change in stromal cell shape (not shown), while HESCs treated with miR-542-3p inhibitor showed a converse transformation. In addition*,* overexpression of miR-542-3p was shown to inhibit HESC proliferation and invasion, and promote apoptosis. It was also shown that there was a high level of miR-542-3p expression in the mouse endometrium on gestation D1, however, it declined rapidly in the decidua by D8 of pregnancy. These results indicate that miR-542-3p might be critical to the regulation of the decidual reaction.

Gene expression is regulated by miRNAs via binding sites within the 3′UTR of the mRNA sequence, which results in changes to the mechanism of post-transcription by either translational repression or mRNA cleavage. To explore the 3′UTR target of miR-542-3p involved in the process of decidualization, we focused on ILK which was predicted by Targetscan and Pictar. ILK is a serine/threonine protein kinase localized within focal adhesions where it acts as a component of a heterotrimer complex, called ILK/PINCH/parvin (IPP) complex, has an important role in connecting integrins to the actin cytoskeleton, and regulates cell matrix interactions^22^. ILK can activate transmembrane signals such as glycogen synthase kinase-3β (GSK-3β) and protein kinase B (PKB/AKT) pathways which enable the regulation of many key biological processes including angiogenesis, cell proliferation, growth, migration, invasion, and tumorigenesis^30,33^. Yen^34^ confirmed the important role of ILK in decidualization of HESCs. In the canonical TGF-β1/SMAD pathway, active TGF-β1 signals through receptor-associated SMADs (R-SMADs); SMAD2 and SMAD3 bind to the cell surface receptor kinases TGF-β1 type I (TβRI) and type II (TβRII) receptors to regulate gene expression. A previous study^14^ suggested that miR-542-3p regulates ILK and its downstream molecules TGF-β1/SMAD2 to suppress oral cancer. In our study, ILK was confirmed to be the direct target of miR-542-3p by the luciferase reporter assay, and RT-PCR and Western blotting results verified that overexpression and inhibition of miR-542-3p functionally regulated ILK and its downstream TGF-β1/SMAD2 expression in HESCs. The restoration study further confirmed that overexpression of ILK attenuated the miR542-3p-induced suppression of cell proliferation and invasion, and promoted cell apoptosis. All these results suggest that the inhibitory role of miR-542-3p on HESC differentiation is through alteration of the expression of the ILK/TGF-β1/SMAD2 signaling pathway.

Angiogenesis is critical for successful embryo implantation and decidualization, while inappropriate endometrial angiogenesis may lead to reproductive failure. In this study, miR-542-3p and ILK are both confirmed to be associated with the regulation of angiogenesis in HESCs. ILK has been shown to be involved in enhancing VEGF expression^35^, and both COX-2 and MMP-9 have been also shown to have a role in mediating angiogenesis by altering VEGF activity^36,37^. In vitro deletion of the ILK gene resulted in suppression of COX-2 and MMP-9. The suppression of COX-2 has been shown to down-regulate the expressions of MMPs in several cancers^38^.We hypothesized that miR-542-3p may be involved in regulating ILK-mediated VEGF, COX-2, and MMP-9 expression in HESCs. Our findings showed that overexpression of miR-542-3p decreased the mRNA and protein expression levels of VEGF, COX-2 and MMP-9. Moreover, assay of tube formation in HUVECs showed that supernatants of miR-542-3p overexpressing HESCs inhibited formation of tubular structures in HUVECs. Our results therefore indicate that miR-542-3p has an inhibitory role in the promotion of tubulogenesis in HUVECs, thereby facilitating angiogenesis in the HESC.

In conclusion, this study reveals that miR-542-3p might play a vital role in decidualization by altering cell characteristics (proliferation, apoptosis and invasion) and angiogenesis via targeting the ILK pathway, which suggests that it might be used as a diagnostic marker for poor endometrial receptivity. However, there are still some limitations in this study. Firstly, although HESC is a well-recognized model used for studying decidualization, we did not investigate the effect of other cells (e.g. epithelial cells) and fetal-maternal interface environment molecules on the decidualization process, the study of which is ongoing. Secondly, the in vivo physiological functions of miR-542-3p and the ILK pathway in decidualization should be employed in-depth in a future study. In addition, further study is warranted in the detection of miR-542-3p in serum samples from patients suffering from recurrent implantation failure to better understand the role of miR-542-3p as a biomarker in early pregnancy.

## Data Availability

All data generated or analysed during this study are included in this published article. The datasets generated during the current study are available from the corresponding author on reasonable request.
